# The Effect of Different Intraperitoneal Hyperthermic Chemotherapy (HIPEC) Regimens on Serum Electrolyte Levels: A Comparison of Oxaliplatin and Mitomycin C

**DOI:** 10.3390/medicina61081345

**Published:** 2025-07-25

**Authors:** Vural Argın, Mehmet Ömer Özduman, Ahmet Orhan Sunar, Mürşit Dinçer, Aziz Serkan Senger, Selçuk Gülmez, Orhan Uzun, Mustafa Duman, Erdal Polat

**Affiliations:** Gastrointestinal Surgery Clinic, Kartal Koşuyolu Yüksek İhtisas Training and Research Hospital, University of Health Sciences, Istanbul 34846, Türkiye; omer_ozduman@yahoo.com (M.Ö.Ö.); orhansunar@hotmail.com (A.O.S.); drmursitdincer@gmail.com (M.D.); serkansenger@yahoo.com (A.S.S.); selcuk.gulmez@sbu.edu.tr (S.G.); orhan.uzun@sbu.edu.tr (O.U.); drmustafaduman@hotmail.com (M.D.); erdal.polat@sbu.edu.tr (E.P.)

**Keywords:** HIPEC, oxaliplatin, mitomycin C, serum electrolytes, colorectal cancer

## Abstract

*Background and Objectives*: This study aimed to compare the effects of HIPEC procedures using oxaliplatin and mitomycin C on serum electrolyte, glucose, and lactate levels, with a specific focus on the carrier solutions employed. *Materials and Methods*: A retrospective analysis was performed on 82 patients who underwent cytoreductive surgery and hyperthermic intraperitoneal chemotherapy (HIPEC) for colorectal peritoneal metastases. Patients were assigned to one of two groups based on the chemotherapeutic agent used: oxaliplatin (*n* = 63) or mitomycin C (MMC, *n* = 19). The oxaliplatin group was further subdivided based on the carrier solution used: 5% dextrose (D5W, *n* = 29) or peritoneal dialysate (*n* = 34). The assignment of regimens was based on institutional protocols and surgeon preference. Pre- and post-HIPEC serum levels of sodium, potassium, bicarbonate, glucose, and lactate were compared. *Results*: Significant biochemical changes were observed across groups, depending on both the chemotherapeutic agent and carrier solution. In the MMC group (peritoneal dialysate), only lactate increased significantly post-HIPEC (*p* = 0.001). In the oxaliplatin–peritoneal dialysate group, significant changes were observed in bicarbonate (*p* = 0.009), glucose (*p* = 0.001), and lactate (*p* < 0.001), whereas sodium and potassium remained stable. The oxaliplatin–D5W group showed significant changes in all parameters: sodium (*p* = 0.001), potassium (*p* = 0.001), bicarbonate (*p* = 0.001), glucose (*p* < 0.001), and lactate (2.4 → 7.6 mmol/L, *p* < 0.001). Between-group comparisons revealed significant differences in sodium, potassium, glucose, and lactate changes (*p* < 0.05), but not in bicarbonate (*p* = 0.099). Demographic and clinical characteristics—including age, sex, primary disease, ICU stay, and 90-day mortality were similar across groups. *Conclusions*: The use of dextrose-containing solutions with oxaliplatin was associated with marked metabolic disturbances, including clinically meaningful hyponatremia, hypokalemia, and hyperglycemia in the early postoperative period. These findings suggest that the choice of carrier solution is as important as the chemotherapeutic agent in terms of perioperative safety. Closer postoperative electrolyte monitoring is recommended when using dextrose-based regimens. The retrospective design and sample size imbalance between groups are acknowledged limitations. Nonetheless, this study offers clinically relevant insights and lays the groundwork for future prospective research.

## 1. Introduction

Peritoneal metastasis is a common manifestation of advanced colorectal cancer (CRC) and is associated with poor prognosis. While systemic chemotherapy remains the standard treatment for metastatic CRC, its efficacy in peritoneal disease is limited. Median overall survival (OS) in patients with isolated peritoneal metastases treated with systemic chemotherapy alone has been reported to be approximately 16.3 months [1]. In contrast, recent randomized controlled trials and meta-analyses suggest that cytoreductive surgery (CRS) combined with hyperthermic intraperitoneal chemotherapy (HIPEC) can significantly improve long-term survival in selected patients, with median OS extending beyond 30 months in some series [2].

The rationale behind CRS-HIPEC lies in the maximal resection of macroscopic disease followed by regional chemotherapy under hyperthermic conditions to eliminate microscopic residual disease [3]. Hyperthermia enhances the cytotoxicity of chemotherapeutic agents, while intraperitoneal administration allows for higher local concentrations with reduced systemic toxicity [4]. Among the most widely used agents in HIPEC are mitomycin C (MMC) and oxaliplatin (OX), both of which have shown oncological efficacy in gastrointestinal malignancies [5].

In addition to the chemotherapeutic agent, the carrier solution plays a critical role in influencing pharmacokinetics and systemic biochemical responses. Commonly used solutions include 5% dextrose (D5W) and peritoneal dialysate, each with distinct osmolarity and ionic content [6]. However, the potential metabolic effects of different agent–carrier combinations, particularly on serum electrolytes and glucose/lactate balance, have not been systematically studied. Considering the known risks of perioperative electrolyte disturbances, hyperglycemia, and acid-base imbalance, understanding these biochemical shifts is essential for treatment safety, postoperative recovery, and patient selection in HIPEC protocols [7]. Yet, few studies in the literature have quantitatively evaluated these outcomes, and none, to our knowledge, have compared multiple agent–carrier combinations in a clinically detailed manner [8].

This study aimed to compare the effects of HIPEC using MMC and OX with different carrier solutions on postoperative serum levels of sodium, potassium, bicarbonate, glucose, and lactate.

## 2. Materials and Methods

### 2.1. Study Design and Patient Selection

This was a retrospective observational study conducted at a single tertiary referral center specializing in peritoneal surface malignancies. Between January 2018 and December 2024, a total of 135 patients aged 18–75 years underwent cytoreductive surgery (CRS) followed by hyperthermic intraperitoneal chemotherapy (HIPEC) for histologically confirmed colorectal peritoneal metastases.

Inclusion criteria were (1) age between 18 and 75 years, (2) Eastern Cooperative Oncology Group (ECOG) performance status 0–2, (3) PCI score ≤ 20, and (4) availability of complete pre- and post-HIPEC laboratory data. Exclusion criteria included (1) use of non-standard HIPEC agents, (2) major intraoperative complications leading to aborted HIPEC, (3) missing biochemical data, and (4) pre-existing severe hepatic or renal dysfunction. 

After applying these criteria, 82 patients were included in the final analysis. Of the excluded 53 patients, 24 received chemotherapeutic agents other than MMC or OX, 19 had incomplete laboratory data, and 10 underwent aborted HIPEC due to unresectable disease.

### 2.2. Treatment Allocation and Surgical Technique

The choice of HIPEC regimen (MMC or OX) and carrier solution (peritoneal dialysate or 5% dextrose [D5W]) was based on institutional protocols, tumor histology, previous systemic treatment, and surgeon preference. All procedures were performed by one of three experienced peritoneal surgeons, following standardized CRS and HIPEC protocols. CRS was performed with the goal of complete macroscopic tumor resection. The extent of disease was assessed intraoperatively using the Peritoneal Cancer Index (PCI), and completeness of cytoreduction (CC) was scored postoperatively. Optimal cytoreduction was defined as CC-0 or CC-1. Following CRS, HIPEC was delivered using the closed-abdomen technique with a dedicated perfusion device. Intraperitoneal temperature was continuously monitored and maintained at 42–43 °C. The perfusate was circulated at a constant flow rate of 1000 mL/min through five inflow and outflow catheters. All patients received standard deep vein thrombosis prophylaxis, urinary catheterization, and intraoperative anesthesia care.

### 2.3. HIPEC Regimens

Mitomycin C (MMC): 30 mg/m^2^, administered intraperitoneally in three divided doses over 90 min using peritoneal dialysis solution as the carrier. Oxaliplatin (OX): 460 mg/m^2^, administered over 30 min in 5% dextrose solution or over 60 min in peritoneal dialysate, combined with intravenous 5-fluorouracil and leucovorin.

### 2.4. Laboratory Analysis and Sampling

Serum electrolyte, glucose, and lactate measurements were obtained at two predefined time points: (1) pre-HIPEC, immediately prior to the initiation of intraperitoneal chemotherapy, and (2) post-HIPEC, immediately following the completion of the perfusion. Serum sodium, potassium, bicarbonate, glucose, and lactate levels were analyzed using the Advia 2120 (Siemens Healthcare Diagnostics, Marburg, Germany) system in a single central institutional laboratory. Laboratory personnel were blinded to the HIPEC regimens. Serum sodium levels were corrected for hyperglycemia using the following standard formula: Corrected Na^+^ = Measured Na^+^ + 0.016 × (Glucose [mg/dL] − 100) [9].

### 2.5. Perioperative Standardization

Intraoperative fluid administration (type and rate of crystalloids, use of albumin) and electrolyte supplementation followed a standardized institutional protocol overseen by a dedicated anesthesiology team. Postoperative ICU care and monitoring were also protocolized.

### 2.6. Statistical Analysis

Descriptive statistics were presented as mean ± SD or median (IQR) as appropriate. Paired *t*-tests or Wilcoxon signed-rank tests were used for within-group comparisons. Between-group comparisons were made using ANOVA or Kruskal–Wallis tests. A post hoc power analysis indicated that the sample size (*n* = 82) provided >85% power to detect clinically significant changes in lactate levels (Cohen’s d = 0.8, α = 0.05). Patients with missing key lab data were excluded from analysis; no imputation was performed. While multivariate adjustments were not feasible due to sample size limitations, baseline characteristics were statistically comparable between groups (Table 1). Group size imbalance (MMC *n* = 19 vs. OX *n* = 63) is acknowledged as a limitation in the discussion.

## 3. Results

Significant differences in serum biochemical alterations were observed between the groups, depending on the chemotherapeutic agent and the type of carrier solution used.

Among patients treated with MMC in peritoneal dialysate, only lactate levels showed a statistically significant post-HIPEC increase (from 2.9 to 4.5 mmol/L, *p* = 0.001), while all other parameters remained within normal physiological ranges and showed no clinically concerning changes (Table 2).

In the oxaliplatin peritoneal dialysate group (*n* = 34), a slight post-HIPEC decrease in sodium levels was observed; however, this was not statistically significant (136.2 → 134.8 mmol/L, *p* = 0.081). Similarly, potassium levels remained stable (3.5 → 3.4 mmol/L, *p* = 0.782). In contrast, bicarbonate levels decreased significantly (20.9 → 20.1 mmol/L, *p* = 0.009), and both glucose (201 → 235 mg/dL, *p* = 0.001) and lactate (2.5 → 3.4 mmol/L, *p* < 0.001) levels increased significantly. Although these changes were statistically significant, most remained within acceptable clinical limits. No intervention was required (Table 3).

In the oxaliplatin D5W group, statistically and clinically significant changes were observed in all parameters: sodium (136.4 → 132.7 mmol/L, *p* = 0.001), potassium (3.7 → 3.3 mmol/L, *p* = 0.001), bicarbonate (21.8 → 19.6 mmol/L, *p* = 0.001), glucose (180.1 → 299.1 mg/dL, *p* < 0.001), and lactate (2.4 → 7.6 mmol/L, *p* < 0.001) (Table 4). In several patients, sodium levels dropped below 130 mmol/L and lactate exceeded 6 mmol/L, potentially indicating metabolic stress and warranting closer postoperative monitoring. 

Following HIPEC, significant alterations were observed in serum biochemical parameters across the overall study population (*n* = 82). Serum sodium levels showed a statistically significant decrease, with a mean reduction of –1.62 mmol/L (95% CI: –2.52 to –0.72, *p* = 0.001). Potassium levels increased slightly but significantly, with a mean change of +0.16 mmol/L (95% CI: +0.03 to +0.29, *p* = 0.017). Bicarbonate concentrations also increased postoperatively (mean Δ = +1.35 mmol/L, 95% CI: +0.77 to +1.93, *p* < 0.001). A substantial elevation in serum glucose was noted, with a mean rise of +59.73 mg/dL (95% CI: +36.46 to +83.00, *p* < 0.001). Similarly, lactate levels increased significantly after HIPEC, with a mean difference of +2.56 mmol/L (95% CI: +1.31 to +3.81, *p* < 0.001). These findings reflect a consistent pattern of biochemical shifts in the early postoperative period (Table 5).

One patient (1/34, 2.9%) in the oxaliplatin–dialysate group died on postoperative day 6 due to septic shock. This patient had a high baseline PCI score (23) and significant comorbidities. Although this represents 100% within that subgroup, it should be interpreted with caution due to the small sample size. There were no statistically significant differences in postoperative morbidity or 90-day mortality among the groups (*p* = 0.81 and *p* = 0.93, respectively). There were no statistically significant differences in grade 3–4 postoperative morbidity (*p* = 0.279) between the three groups. Overall, the three groups were comparable in demographic and clinical characteristics, including age, sex, primary tumor location, ICU stay duration, and comorbidity burden (Table 1 and Table 6).

## 4. Discussion

This study retrospectively investigated the biochemical effects of different HIPEC regimens, specifically oxaliplatin (OX) and mitomycin C (MMC), administered with distinct carrier solutions. Rather than merely documenting changes in electrolyte and metabolite levels, these findings underscore the clinical and pharmacological implications of carrier solution choice during HIPEC, which is often overlooked. The results suggest that carrier solutions do not merely serve as passive vehicles but actively influence systemic homeostasis and patient safety profiles.

Patients receiving OX in 5% dextrose (D5W) exhibited substantial biochemical derangements, including significant hyponatremia, hypokalemia, hypobicarbonatemia, and hyperglycemia, suggesting a metabolically unstable profile. In contrast, those treated with OX in peritoneal dialysis solution showed a more moderate biochemical response, while the MMC group displayed the most stable biochemical profile, with only a significant rise in lactate. These observations highlight the underappreciated role of carrier solutions in modulating postoperative physiology. D5W, although frequently used with oxaliplatin due to solubility requirements, is a hypotonic and glucose-rich medium that can disrupt electrolyte balance via dilutional effects and osmotic shifts [10]. Hypotonicity promotes hyponatremia, while elevated glucose levels may lead to osmotic diuresis, hyperglycemia, and, when combined with bicarbonate loss, a metabolic acidosis-like state [11,12,13]. In our study, the OX + D5W group exhibited a marked increase in lactate (to 7.6 mmol/L) and a simultaneous drop in bicarbonate, indicating a possible shift toward metabolic acidosis. Although this did not require emergent intervention, such changes may necessitate closer ICU monitoring or bicarbonate supplementation in at-risk patients [14]. In contrast, MMC was associated with minimal electrolyte disturbance. The mild rise in lactate levels in the MMC group likely reflects surgical stress rather than the chemotherapeutic agent or carrier solution. Our findings align with prior reports that glucose-free solutions like peritoneal dialysate, when combined with MMC, yield more stable metabolic profiles [15,16].

From a pharmacokinetic perspective, the carrier fluid influences both drug absorption and systemic physiology. Sodium losses due to dilution or peritoneal exudation, potassium shifts due to intracellular migration, and bicarbonate depletion due to renal and metabolic stress are all exacerbated by hyperosmolar or hypotonic carrier media. These metabolic shifts are not benign: they are associated with neurologic symptoms, delayed recovery, increased infection risk, and even prolonged hospitalization if not addressed proactively [17,18].

While most patients tolerated these shifts without clinical deterioration, one patient in the oxaliplatin + dialysate group died on postoperative day 6 from septic shock. This individual had high baseline PCI and significant comorbidities. Although this case is an outlier, it highlights the importance of early recognition and intervention in metabolically fragile patients [19].

In light of these findings, standardized perioperative monitoring of electrolytes, glucose, and lactate should be a routine component of HIPEC protocols, especially when using D5W. Furthermore, future studies should explore strategies to mitigate these imbalances, including prophylactic sodium or bicarbonate supplementation, use of alternative isotonic carriers, or pre-treatment correction of underlying metabolic risks.

Finally, the retrospective and single-center design of this study limits causal inference. Future prospective, randomized trials comparing HIPEC agent–carrier combinations are warranted to confirm these findings and inform protocol optimization.

## 5. Conclusions

This study demonstrated that the type of carrier solution used during HIPEC significantly affects serum electrolyte and metabolite levels, particularly when combined with oxaliplatin. The use of 5% dextrose as a carrier was associated with marked alterations, including hyponatremia, hypokalemia, hypobicarbonatemia, hyperglycemia, and elevated lactate levels, some of which may indicate a shift toward metabolic acidosis. In contrast, MMC combined with peritoneal dialysate resulted in a more stable biochemical profile. These findings highlight the importance of selecting carrier solutions not only based on drug compatibility, but also on their physiological safety.

From a clinical perspective, patients receiving oxaliplatin in glucose-containing solutions may require closer postoperative monitoring of serum electrolytes, glucose, and acid–base balance, especially in those with pre-existing metabolic vulnerability. Standardized perioperative protocols, including early biochemical screening and fluid management strategies, may help mitigate potential complications. Prospective, randomized studies are warranted to validate these findings and to identify optimal agent–carrier combinations that ensure both oncological efficacy and metabolic stability in HIPEC.

### Limitations

The retrospective design of our study and the limited number of patients in some subgroups may restrict the generalizability of the results. Future prospective and randomized studies should investigate the metabolic effects of different chemotherapeutic agents and carrier solution combinations in larger patient populations. Furthermore, the clinical implications of these biochemical changes, such as their impact on complication rates, recovery time, and overall survival, should also be evaluated. In addition, we did not routinely measure serum calcium, magnesium, or phosphorus levels in the postoperative period, which is a limitation. These parameters are monitored in many centers, and their inclusion in future studies may provide further insight into the metabolic consequences of HIPEC.

## Figures and Tables

**Table 1:** Baseline demographic and clinical characteristics of patients.

	Oxaliplatin (*n* = 16)	Mitomycin C (*n* = 19)	*p* Value
**Age (years)**			0.520
Mean	54.2	53.8	
Median	56.5	58	
Range	24–70	25–70	
**Gender [*n*, (%)]**			0.235
Male	32 (51.6%)	8 (40%)	
Female	30 (48.4%)	12 (60%)	
**Primary disease [*n* (%)]**			0.06
Colorectal cancer	46 (74.2%)	11 (55%)	
Psödomiksoma peritonei	16 (25.8%)	9 (45%)	
**Intensive care unit stay (days)**			0.392
Mean	1.2	1.4	
Median (range)	1 (1–5)	1 (1–5)	

Data are presented as mean ± standard deviation or *n* (%). *p*-values were calculated using *t*-test, chi-square, or Mann–Whitney U test as appropriate.

**Table 2:** Changes in serum electrolytes before and after HIPEC with mitomycin C in peritoneal dialysate (*n* = 19).

Electrolytea	Pre-HIPEC Average	Post-HIPEC Average	*p*-Value	Clinically Significant
Sodium (mmol/L)	139.5	136.4	0.936	Within normal range
Potassium (mmol/L)	3.5	3.5	0.755	Stable
Bicarbonate (mmol/L)	20.7	21.8	0.219	Slight improvement
Glucose (mmol/L)	182.7	180.1	0.168	Remained stable
Lactate (mmol/L)	2.9	4.5	**0.001**	Mild elevation but not critical

Lactate levels > 4.0 mmol/L, glucose > 180 mg/dL, and sodium < 135 mmol/L were considered clinically abnormal. In the oxaliplatin + D5W group, several patients exceeded these thresholds post-HIPEC.

**Table 3:** Changes in serum electrolytes before and after HIPEC with oxaliplatin in peritoneal dialysate (*n* = 34).

Electrolytea	Pre-HIPEC Average	Post-HIPEC Average	*p* Value	Clinically Significant
Sodium (mmol/L)	138.1	137.4	0.081	Mild decrease, still within range
Potassium (mmol/L)	3.4	3.3	0.782	Borderline hypokalemia
Bicarbonate (mmol/L)	20.9	20.1	**0.009**	Borderline low
Glucose (mmol/L)	201	235.5	**0.001**	Marked hyperglycemia
Lactate (mmol/L)	2.5	3.4	**<0.001**	Moderately elevated

Lactate levels > 4.0 mmol/L, glucose > 180 mg/dL, and sodium < 135 mmol/L were considered clinically abnormal. In the oxaliplatin + D5W group, several patients exceeded these thresholds post-HIPEC.

**Table 4:** Changes in serum electrolytes before and after HIPEC with oxaliplatin in D5W (*n*= 29).

Electrolytea	Pre-HIPEC Average	Post-HIPEC Average	*p* Value	Clinically Significant
Sodium (mmol/L)	136.4	132.7	**0.001**	Mild hyponatremia in several patients
Potassium (mmol/L)	3.7	3.3	**0.001**	Borderline hypokalemia
Bicarbonate (mmol/L)	21.8	19.6	**0.001**	Mild metabolic acidosis in some cases
Glucose (mmol/L)	180.1	299.1	**<0.001**	Marked hyperglycemia
Lactate (mmol/L)	2.4	7.6	**<0.001**	Lactate > 4 mmol/L in multiple patients

Lactate levels > 4.0 mmol/L, glucose > 180 mg/dL, and sodium < 135 mmol/L were considered clinically abnormal. In the oxaliplatin + D5W group, several patients exceeded these thresholds post-HIPEC.

**Table 5:** Comparison of group-based changes in serum electrolyte and metabolite level.

Parameters	Δ Mean	SD	95% Confidence Interval	Overall Significance (Kruskal–Wallis)	Significant Pairwise Group Differences (Bonferroni)	*p*-Value
Δ Sodium	−1.62	4.16	−2.52 to −0.72	Yes	Group 2 vs. Group 3	**0.043**
					Group 2 vs. Group 1	**0.006**
Δ Potassium	0.16	0.58	0.03 to 0.29	Yes	Group 2 vs. Group 1	**0.012**
					Group 2 vs. Group 3	0.054
Δ Bicarbonate	1.35	2.69	0.77 to 1.93	No	—	0.099
Δ Glucose	59.73	107.52	36.46 to 83	Yes	Group 2 vs. Group 1	**0.015**
					Group 2 vs. Group 3	**0.047**
Δ Lactate	2.56	5.78	1.31 to 3.81	Yes	Group 3 vs. Group 2	**<0.001**

Note: “Δ” indicates the change in parameter levels before and after HIPEC. Group 1 = mitomycin C + peritoneal dialysate, Group 2 = oxaliplatin + D5W, Group 3 = oxaliplatin + peritoneal dialysate. Statistical comparisons were performed using Kruskal–Wallis tests followed by Bonferroni-adjusted pairwise comparisons. “Yes” indicates a statistically significant overall change. “No” indicates no statistically significant change.

**Table 6:** Postoperative Clavien–Dindo grade 3–4 morbidity and 90-day mortality rates by groups.

Groups	Clavien–Dindo 3–4 Morbiditiy (*n*, %)	90-Day Mortality (*n*, %)
Mitomycin C + peritoneal dialysate (*n* = 19)	4 (21.1%)	0 (0%)
Oxaliplatin + D5W (*n* = 29)	10 (34.5%)	0 (0%)
Oxaliplatin + peritoneal dialysate (*n* = 34)	6 (17.6%)	1 (100%)

There was no statistically significant difference in Clavien–Dindo grade 3–4 morbidity among the groups (chi-square, *p* = 0.279). Similarly, no significant difference in 90-day postoperative mortality was observed (Fisher’s exact test, *p* = 0.489).

## Data Availability

No new data were created or analyzed in this study.
